# Retinal Glycoprotein Enrichment by Concanavalin A Enabled Identification of Novel Membrane Autoantigen Synaptotagmin-1 in Equine Recurrent Uveitis

**DOI:** 10.1371/journal.pone.0050929

**Published:** 2012-12-07

**Authors:** Margarete E. Swadzba, Stefanie M. Hauck, Hassan Y. Naim, Barbara Amann, Cornelia A. Deeg

**Affiliations:** 1 Institute of Animal Physiology, Department of Veterinary Sciences, Ludwig-Maximilians University, München, Germany; 2 Research Unit for Protein Science, Helmholtz Zentrum München–German Research Center for Environmental Health (GmbH), Neuherberg, Germany; 3 Department of Physiological Chemistry, University of Veterinary Medicine Hannover, Hannover, Germany; Université Paris Descartes, France

## Abstract

Complete knowledge of autoantigen spectra is crucial for understanding pathomechanisms of autoimmune diseases like equine recurrent uveitis (ERU), a spontaneous model for human autoimmune uveitis. While several ERU autoantigens were identified previously, no membrane protein was found so far. As there is a great overlap between glycoproteins and membrane proteins, the aim of this study was to test whether pre-enrichment of retinal glycoproteins by ConA affinity is an effective tool to detect autoantigen candidates among membrane proteins. In 1D Western blots, the glycoprotein preparation allowed detection of IgG reactions to low abundant proteins in sera of ERU patients. Synaptotagmin-1, a Ca2+-sensing protein in synaptic vesicles, was identified as autoantigen candidate from the pre-enriched glycoprotein fraction by mass spectrometry and was validated as a highly prevalent autoantigen by enzyme-linked immunosorbent assay. Analysis of Syt1 expression in retinas of ERU cases showed a downregulation in the majority of ERU affected retinas to 24%. Results pointed to a dysregulation of retinal neurotransmitter release in ERU. Identification of synaptotagmin-1, the first cell membrane associated autoantigen in this spontaneous autoimmune disease, demonstrated that examination of tissue fractions can lead to the discovery of previously undetected novel autoantigens. Further experiments will address its role in ERU pathology.

## Introduction

In life sciences, proteomics has become a well established and extremely valuable research tool over the years, helpful in exploring and understanding not only physiological processes, but also pathogenesis of a variety of diseases on a molecular level. Especially in the field of autoimmune diseases, researchers can profit immensely from employing proteomic methods [1], [2], [3].

Equine recurrent uveitis (ERU) is an organ specific autoimmune disease characterized by remitting-relapsing episodes of intraocular inflammation and is an established spontaneous animal model for its human counterpart autoimmune uveitis (AU) [1]. While causative factors and exact pathogenesis of ERU are still unknown, proteomic methods provided helpful insights into different aspects of the disease. Differential proteome analyses were successfully applied through comparing target tissue or immune proteomes of controls and ERU cases. Recently, we deciphered changed protein expression in innate immune cells of ERU affected individuals [2]. Another important area of application is the continuous search for autoantigens, where proteomic identification methods led to a gradual expansion of knowledge about the ERU autoantigen spectrum [3], [4].

Binding of autoantibodies to self-antigens is an important aspect of many autoimmune diseases, including ERU [5]. In order to understand pathological mechanisms and use this knowledge to a patient's advantage, it is of paramount importance to know which players are involved. Autoimmune attacks have grave consequences for the functionality of the targeted structure, which is, in case of ERU, the retina. Completing our knowledge of the targeted autoantigen spectrum, which broadens as the disease progresses [5], would further enable us to understand heterogeneous clinical manifestations. A challenge in this task, however, is the presence of several sub-groups of proteins which might slip through the reading frame of an experiment, e.g. due to low abundance in the examined tissue. Investigating such sub-groups or fractions of tissue proteins in detail for potential autoantigens often requires an adaptation of the standard experimental setup in order to shift the focal point of the experimental reading frame in the right direction. Glycoproteins are conceivably a highly interesting fraction of retinal proteins to explore in search for novel autoantigen candidates, as post-translationally modified proteins are already known to be autoantibody targets in several autoimmune diseases [6], [7] and glycosylation is the most frequent post-translational modification [8]. Further, glycoproteins are predominantly present on cell interfaces as membrane proteins and are involved in a large number of cell-cell interactions [9]. Location of glycoproteins on cellular membranes makes them especially interesting as autoantigens, because one expects to have a cell membrane autoantigen targeted initially in autoimmune diseases. Breakdown of blood-retinal barrier and subsequent infiltration of the inner eye with autoreactive immune cells is a crucial mechanism in ERU [10], [11], [12]. Initiating events are expected to take place at cell membranes as particularly exposed barrier structures, but so far no membrane protein was identified as autoantigen in ERU or autoimmune uveitis of man or in experimental autoimmune uveitis models [13], [14]. An impressive example for the impact of the discovery of membrane-bound autoantigens was the very recent identification of Aquaporin-4 (AQP4), which is the principal cellular water channel of astrocytes, as autoantigen in neuromyelitis optica [15], [16]. Its identification was a crucial leap forward in this research field, leading to substantial progress in understanding the pathogenesis of neuromyelitis optica and enabling discrimination of patients with neuromyelitis optica from those with multiple sclerosis [15], [16], [17]. This discovery of AQP4 demonstrated that ongoing search for novel autoantigens in autoimmune diseases is not only a matter of completing the picture, but should be continued and intensified by all means, as its results can have profound effect on clinical diagnosis. Interestingly, AQP4 is also linked to intraocular inflammation, as AQP4 and Kir4.1, the two main channel proteins in retinal Müller glia cells are differentially regulated during ocular inflammation [18], [19]. AQP4 in retinal Müller glia cells also plays a role in ERU, where it is upregulated in uveitis retinas, but shows a disclocation to the outer nuclear layer in its expression pattern [20].

The aim of the study conducted here was to detect cell membrane autoantigens in ERU, using a pre-enriched retinal glycoprotein preparation as target. Our approach was based on pre-enriching glycoproteins from retinal tissue via affinity to Concanavalin A (ConA), which is a lectin widely used in glycoprotein enrichment and isolation techniques, especially known for its broad selectivity [21], [22]. This initial enrichment step was followed by a combination of proteomic methods, including Western blot and mass spectrometry and the subsequent successful validation of a candidate protein identified from the pre-enriched glycoprotein fraction.

## Materials and Methods

### Retina specimen and serum samples

For this study, organic material derived from 67 healthy horses and 134 ERU cases was examined. In the screening approach by Western blots, sera from 17 eye-healthy horses and 17 ERU cases were used. ELISA validation was performed with a serum cohort of 38 eye-healthy controls and 106 ERU cases.

Retinal tissue samples from 5 healthy and 7 ERU affected eyes were used for Western blot quantifications, retinal sections from 7 healthy and 7 ERU affected eyes were analyzed by immunohistochemistry.

Eyes providing healthy porcine and healthy equine retinal samples were obtained from animals slaughtered at a local abattoir. ERU affected eyes derived from horses that were diagnosed with ERU and had to be enucleated during a therapeutical procedure. Porcine retinal samples were used for glycoprotein enrichment. Equine retinal samples were used in Western blot quantification and immunohistochemistry. Retina specimens for proteomic experiments were prepared as previously described [23]. Eyes used for healthy and ERU-affected retinal sections in immunohistochemistry were prepared according to a standardized protocol [24]. Eyes were considered normal based on the diagnosis of the veterinarian present at the abattoir, medical histories of the horses as provided by their owners, and preliminary histological analysis. All ERU cases were patients diagnosed with ERU at the Equine Clinic of LMU Munich, Munich, Germany.

### Ethics statement

No experimental animals were used in this study. Horses were treated according to the ethical principals and guidelines for scientific experiments on animals according to the ARVO statement for the use of animals in Ophthalmic and Vision research. Asservation of blood samples was permitted by the local authority, Regierung von Oberbayern (Permit number: AZ 55.2-1-54-2532.3-21-12). Blood samples were collected for purposes of scientific research with permission from the Equine Clinic of LMU Munich, Munich, Germany. The collection and use of equine and porcine eyes derived from animals that were killed due to a research-unrelated cause, was approved for purposes of scientific research by the appropriate board of the veterinary inspection office Munich, Germany (Permit number: 8.175.10024.1319.3). Equine eyes were obtained with permission at Pferdemetzgerei Veit, Deggendorf, Germany. Porcine eyes were obtained with permission at Münchner Schlachthof Betriebs GmbH, Department of pig slaughtering, Munich, Germany.

### Enrichment of retinal glycoproteins

For retinal glycoprotein enrichment, we followed a protocol based on a previously described method [22]. We used Concanavalin-A (ConA) coupled sepharose beads (ConA Sepharose 4B, GE Healthcare, Freiburg, Germany), binding buffer (20 mM TrisHCl, 0.5 M NaCl, 1 mM Ca^2+^, 1 mM Mn^+^, pH 7) and elution buffer (20 mM TrisHCl, 500 mM D-mannose). Briefly, ConA coupled sepharose beads were portioned to 500 µL and washed four times with 1.5 ml binding buffer. Twelve healthy retinas (derived from slaughtered pigs) were snap frozen in liquid nitrogen and mechanically crushed. Subsequently, 500–750 µL corresponding to 7.5 to 11 mg protein of retinal homogenate dissolved in binding buffer were incubated with each portion of ConA-sepharose beads, under agitation for 2 hours at 4°C. Next, non-bound fractions were removed by washing each portion four times with binding buffer. Afterwards, the ConA affine fraction was eluted with 1 mL elution buffer per portion under agitation for 2 hours at 4°C. Glycoprotein-depleted and glycoprotein-enriched eluates were precipitated with three fold volumes of ethanol, at −20°C over night. Precipitated proteins were collected by centrifugation for 20 minutes, at 4°C with 10000× g and dry pellets were stored frozen until further processing.

### 1D SDS Page and Western blot screening

Retinal whole lysate, glycoprotein-depleted and glycoprotein-enriched pellets were solubilized in lysis buffer (9M urea, 2 M thiourea, 4% CHAPS, 1% DTT), and protein content was quantified with Bradford assay (Sigma-Aldrich, Deisenhofen, Germany). Proteins were resolved in 1D SDS gels, then stained with colloidal Coomassie or blotted semidry onto polyvinylidene difluoride membranes (GE Healthcare) for Western blot experiments. In Western blot screenings, unspecific binding was blocked with 1% polyvinylpyrrolidone in PBS with 0.05% Tween20 (PBS-T). Blots were incubated with horse serum as primary antibody source (healthy or ERU cases, dilution 1∶1000) overnight at 4°C. After washing, blots were incubated with peroxidase-coupled (POD) secondary antibody (goat anti horse IgG Fc-POD, dilution 1∶10000; Biozol, Eching, Germany). Signals were detected by enhanced chemiluminescence (ECL) on x-ray films (Christiansen, Planegg, Germany).

### Mass spectrometry

LC-MS/MS mass spectrometry was performed as previously described [25]. Briefly, the candidate protein band was excised from the blot membrane and subjected to on-membrane tryptic digest. Resulting peptides were separated on a reversed phase chromatography column (PepMap, 15 cm×75 µm ID, 3 µm/100A pore size, LC Packings), which was operated on a nano-HPLC (Ultimate 3000, Dionex, Idstein, Germany) connected to a linear quadrupole ion-trap Orbitrap (LTQ Orbitrap XL, Thermo Fisher Scientific, Schwerte, Germany). The mass spectrometer was operated in the data-dependent mode to automatically switch between Orbitrap-MS and LTQ-MS/MS acquisition. Survey full scan MS spectra (from m/z 300 to 1500) were acquired in the Orbitrap resolution R = 60,000 at m/z 400 and up to ten most intense ions were selected for fragmentation on the linear ion trap using collision induced dissociation at a target value of 100,000 ions and subsequent dynamic exclusion for 30 seconds. MS/MS spectra were used for peptide identification with Mascot (version 2.3.02, Matrix Science, London, UK; http://www.matrixscience.com) (search parameters: carbamidomethylation set as fixed modification, oxidation of methionines and deamidation of asparagine and glutamine set as variable modifications, parent ion tolerance restricted to 10 ppm and fragment ion tolerance to 0.6 Da) in the Ensembl database for pig (Sus scrofa; Sscrofa10.2.67.pep, downloaded from ftp://ftp.ensembl.org/pub/current_fasta/sus_scrofa/pep/). Results were imported into Scaffold (version 3.4.3; Proteome Software) and protein identification probability threshold was set to 95%, peptide threshold to 80% with a minimum of two peptides identified per protein.

### Enzyme-linked immunosorbent assay (ELISA)

Polystyrene 96-well flat-bottomed plates (Nunc Maxisorb; Fisher Scientific GmbH, Schwerte, Germany) were coated with full-length recombinant synaptotagmin-1 (Acris, Herford, Germany). For coating, protein was diluted at 1 µg/mL in NaHCO_3_ buffer (pH 9.6). Each well was incubated with 100 µL of this dilution overnight at 4°C. To prevent nonspecific binding, plates were blocked with 200 µL per well of 0.5% gelatin at 37°C for 1 hour. Serum samples derived from healthy horses and ERU cases were used as primary antibody source, (dilution 1∶1000, in PBS-T), and the wells were incubated at 37°C for 1 hour (100 µL/well) and washed with PBS-T (three times, 300 µL/well). Wells were incubated with goat anti-horse IgG Fc POD (dilution 1∶50000; Biozol, Eching, Germany) as secondary antibody (50 µL/well for 1 hour at 37°C). Using tetramethylbenzidine (Sigma-Aldrich) as a substrate, absorbance (OD) at 450 nm was measured with a microplate reader after stopping the reaction with 1 M sulfuric acid (50 µL/well). Each plate contained wells in which primary antibody incubation was omitted to enable later correction for blank values, and in addition wells incubated with negative control samples and positive control samples with were determined by Western blot and confirmed in a preliminary ELISA experiment with identical setup. The cutoff was set at a tenfold increased SD above the average OD of negative control samples. Absorbance values above the determined cutoff value were counted as positive. Numbers of positive reactions in control samples and ERU samples were compared by applying the ChiSquare test; differences were considered significant at p≤0.05. Statistical analysis was performed with GraphPad Prism 5.04 software (Statcon, Witzenhausen, Germany). Prevalence in % was calculated by dividing the number of positive sera by the number of tested sera for each group.

### Quantification of Syt1 expression with Western blots

Equal total protein amounts of retinal samples derived from 5 healthy controls and 7 ERU cases were loaded onto SDS Gels, blotted, blocked and developed with ECL as already described in section 3.2. For detection of Syt1, blots were incubated with polyclonal rabbit anti-synaptotagmin-1 antibody (dilution 1∶1000, Abcam, Berlin, Germany) followed by goat anti rabbit IgG POD (dilution 1∶3000, Sigma-Aldrich). Western blots signals were imaged on a transmission scanner using LabScan 5.0 software and quantified by densitometry with ImageQuantTL software (all GE Healthcare). Signals were normalized to beta-actin content after staining of lanes with monoclonal mouse anti beta-actin antibody (dilution 1∶5000, Sigma-Aldrich) prior to statistical analysis. As Kolmogorov-Smirnov test showed that data were not distributed normally (p≤0.05), Mann-Whitney test was applied for calculation of statistical significance (p≤0.05). Statistical analysis was performed using Paleontological Statistics software (PAST, http://folk.uio.no/ohammer/past/index.html).

### Immunohistochemistry

Posterior eyecups were fixed in Bouin's solution (Sigma-Aldrich) as previously described [20]. The resulting tissue blocks [24] were sectioned to 8 µm and mounted on coated slides (Superfrost, Menzel, Braunschweig, Germany). Heat antigen retrieval was performed at 99°C for 15 minutes in 0.1 M EDTA-NaOH buffer (pH 8.0). To prevent nonspecific antibody binding, sections were blocked with 1% bovine serum albumin (BSA) in Tris-buffered saline containing 0.05% Tween20 (TBS-T) and 5% goat serum for 40 minutes at RT for 3 h at room temperature. Using polyclonal rabbit anti-synaptotagmin-1 (dilution 1∶1500; Abcam) and monoclonal mouse anti Glucose-regulated protein 78 (GRP78) antibody (dilution 1∶50, BD Biosciences, Heidelberg, Germany), primary antibody incubation was performed over night. Sections were incubated with appropriate Alexa Fluor-labeled secondary IgG antibodies for 30 minutes at room temperature. For anti-synaptotagmin-1, goat anti-rabbit IgG alexa647 was used, for anti GRP78, we used goat anti-mouse IgG coupled to alexa546 (dilution 1∶500; all from Invitrogen, Karlsruhe, Germany). All antibodies were diluted in TBS-T containing 1% BSA. Cell nuclei were counterstained with DAPI (dilution of 1∶1000; Invitrogen). As last step, sections were mounted with glass coverslips using fluorescent mounting medium. Fluorescent images were recorded with Axio Imager M1 or Z1 (Zeiss, Göttingen, Germany) and the Axio Vision 4.6 software (Zeiss).

## Results

### Retinal glycoproteins enriched by ConA affinity are suitable for 1D Western blot screening

**Figure 1:**
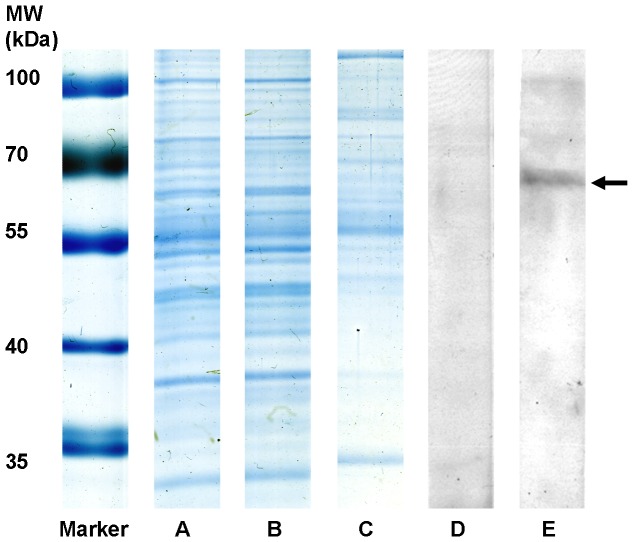
Retinal glycoprotein preparation in 1D SDS-Gel, stained with colloidal Coomassie and in 1D Western blot. (MW): Molecular weight marker, (A) retinal whole-tissue lysate, untreated, (B) retinal glycoprotein-depleted fraction after lectin affinity chromatography, (**C**) retinal glycoprotein fraction enriched by lectin affinity. Similar staining patterns of lanes A and B as opposed to different band pattern in lane C indicate a successful enrichment of normally low-abundant glycoproteins. (D) Representative 1D Western blots of glycoprotein-enriched retinal fractions, incubated with healthy control serum showing no reaction to retinal glycoproteins, (E) and incubated with serum of an ERU case that reacted to candidate protein band (arrow).

Retinal glycoproteins were pre-enriched by taking advantage of lectin affinity, following a protocol based on a method previously described by Naim and Koblet [22]. Briefly, ConA-coupled sepharose beads were incubated with retinal tissue. While the non-glycoprotein retinal fraction, which did not bind to ConA was removed, glycoproteins bound to the beads due to their affinity to ConA and were retrieved by elution with D-mannose. For control of enrichment efficacy, we stained protein patterns of unfractionated retinal tissue, retinal glycoprotein-depleted fraction and glycoprotein-enriched fractions after separation with SDS gels. Gels were stained with colloidal Coomassie blue and compared based on differential staining patterns and intensities (Figure 1, A–C). While staining patterns of unfractionated retinal tissue (Fig. 1A) and glycoprotein-depleted fraction (Fig. 1B) were quite similar, the glycoprotein-enriched fraction (Fig. 1C) clearly differed from these two. This indicated that the bulk of high abundant, non-glycosylated proteins was left behind in the unbound fraction and we had successfully enriched glycoproteins from retinal tissue. Since many protein bands that were stained in the glycoprotein fraction (Fig. 1C), were not visible in retinal whole lysate (Fig. 1A) although equal amounts of total protein were loaded, we conclude that many of these proteins would not have been detected without enrichment due to their low abundance. Also, although the fraction of enriched proteins consisted of low abundant proteins, the great variety of bands in this fraction (Fig. 1C) demonstrated that we achieved better access to not only a few, but a fairly large number of potential autoantigens which were previously undetectable. Retinal glycoprotein preparation was then tested for IgG specific autoimmune reactions in 1D Western Blots. Sera from healthy controls and ERU cases were tested for autoreactive immune responses to the glycoprotein fraction (Fig. 1D healthy, Fig. 1E ERU). A protein band of a MW slightly less than 70 kDa, which was not bound by any control samples, was clearly bound by ERU IgG (Fig. 1E). Subsequently, this protein band was clearly identified with mass spectrometry as synaptotagmin-1 (Table 1). Synaptotagmin-1 (abbrev. Syt1) is a N-glycosylated protein with a crucial role in the Ca^2+^-triggered exocytosis of synaptic vesicles [26], [27], reflecting the N-type glycoprotein affinity of ConA, the lectin used in the pre-enrichment step. Gene ontology annotations for the cellular location of this protein included synaptic vesicle membrane and plasma membrane (source: UniProt Database, Accession number P21579). As Syt1 was never identified as ERU autoantigen candidate in previous studies, this experiment demonstrated that the enrichment efficacy of the applied method was sufficient to enable a detection of serum IgG reactions specific for this protein fraction.

**Table 1:** Candidate glycoprotein band as identified by Liquid chromatography-mass spectrometry/mass spectrometry (LC-MS/MS).

Protein name^A^	Accession number^B^	Gene name^C^	Peptide count^D^
Synaptotagmin-1	ENSSSCP00000001015	SYT1	3

(A) : Protein name: Name of the identified protein.

(B) Accession number as listed in Ensembl database (http://www.ensembl.org),

(C) Gene name as listed in HGNC database (http://www.genenames.org/),

(D) Number of peptides the protein was identified with. Syt1 was identified with an identification probability of 100%.

### Prevalence of serum IgG specific for SYT1 significantly higher in ERU group

**Figure 2:**
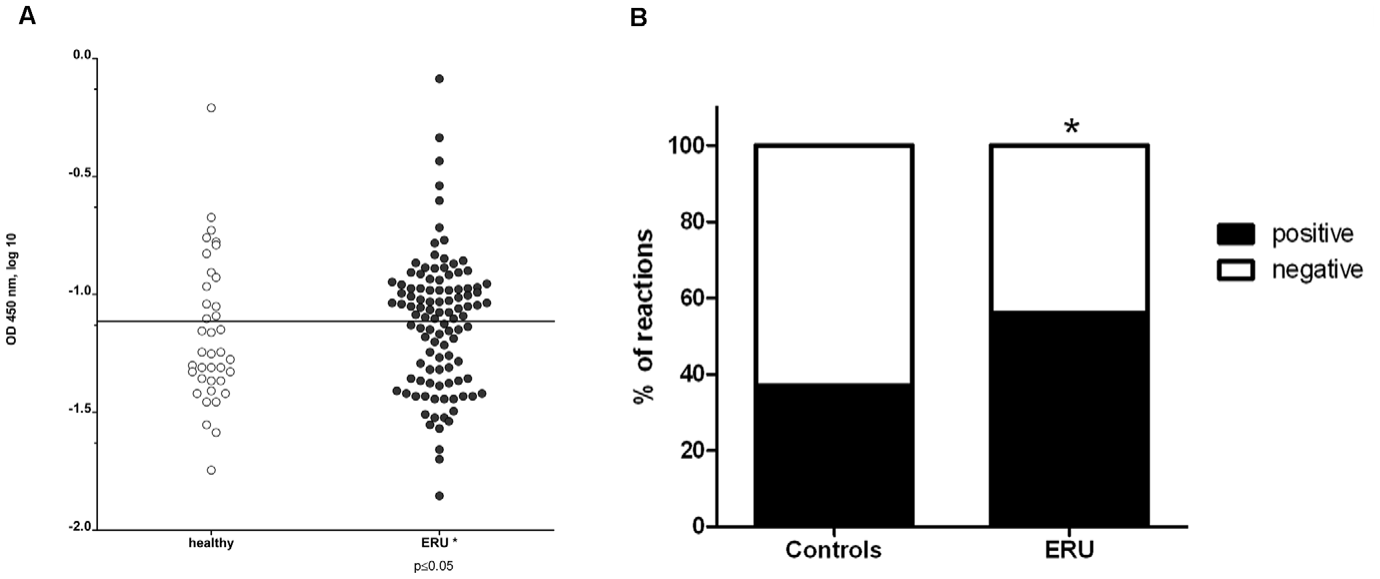
Prevalence of anti Syt1 sera IgG autoantibodies detected by indirect ELISA. Figure 2A: Anti-synaptotagmin-1 positive IgG in healthy sera (left, white dots) and ERU cases (right, black dots). Each dot represents the absorbance value of an individual serum sample, measured at 450 nm). The Cut-off value was set at the mean value of negative controls plus the 10-fold standard deviation, represented by the horizontal separation line. In ERU samples, anti-Syt1-autoantibodies are more frequently present than in healthy samples (* = p≤0.05). Figure 2B: Comparison of positive reactions (black) and negative reactions (white) in control sera (left bar) and ERU samples (right bar) expressed in per cent. While in controls, the prevalence of positive IgG reactions to Syt1 was 37%, prevalence in the ERU group was 56%. Prevalence in percent was calculated by dividing numbers of positive samples by numbers of tested samples in each group.

In order to verify our finding from the proteomics experiment and validate our novel glycoprotein autoantigen Syt-1, we performed an indirect Enzyme-linked immunosorbent assay (ELISA) to detect anti Syt1 autoantibodies using purified Syt1 as a target. This experiment provided us with two important results (Figs. 2A and 2B): first, Syt1 was specifically targeted by autoreactive IgGs (Fig. 2A) and second, the prevalence of Syt1 autoantibodies in the ERU group was 56% (Fig. 2B). Comparing the prevalence of Syt1 specific autoantibodies between ERU cases (Fig. 2A, right, black dots) and healthy controls (Fig. 2B, left, white dots), we found a significantly higher occurrence of Syt1 autoantibodies in ERU group (p≤0.05). ELISA results illustrating the reaction behavior of all individual serum samples are presented in Figure 2.

### Reduced Syt1 expression in ERU affected retinas

**Figure 3:**
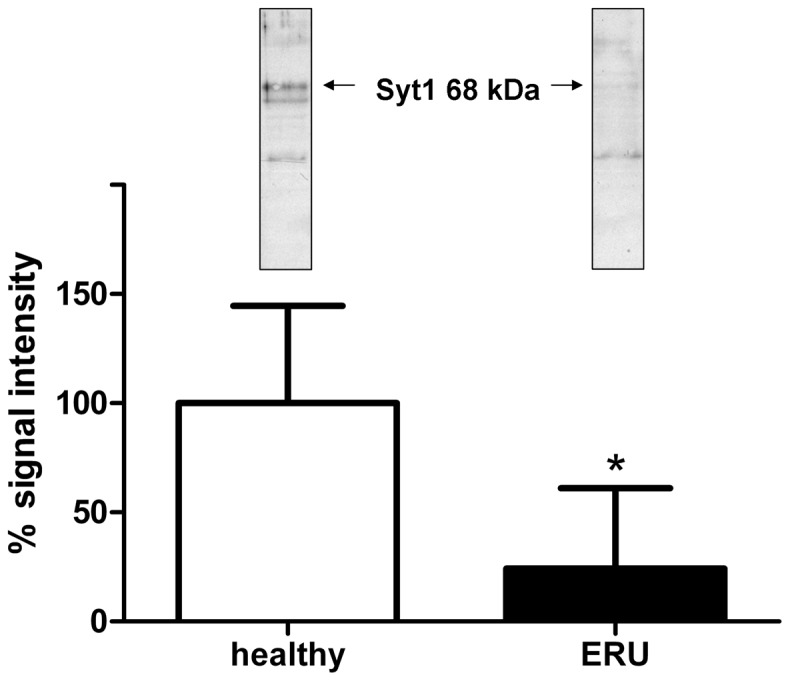
Unequal Syt1 expression in healthy and ERU affected retinal tissues. Western blot quantification of Syt1 expression in healthy retinas (white bar, n = 5) and ERU (black bar, n = 7). Representative Syt-1 blots for each group are inserted above respective bars (left, healthy and right, ERU). The band at 68 kDa was used for quantification (arrows). Mean signal intensity of control samples was set as a 100%, mean signal intensity in ERU samples was significantly reduced to 24% (±34%) (* = p≤0.05).

**Figure 4:**
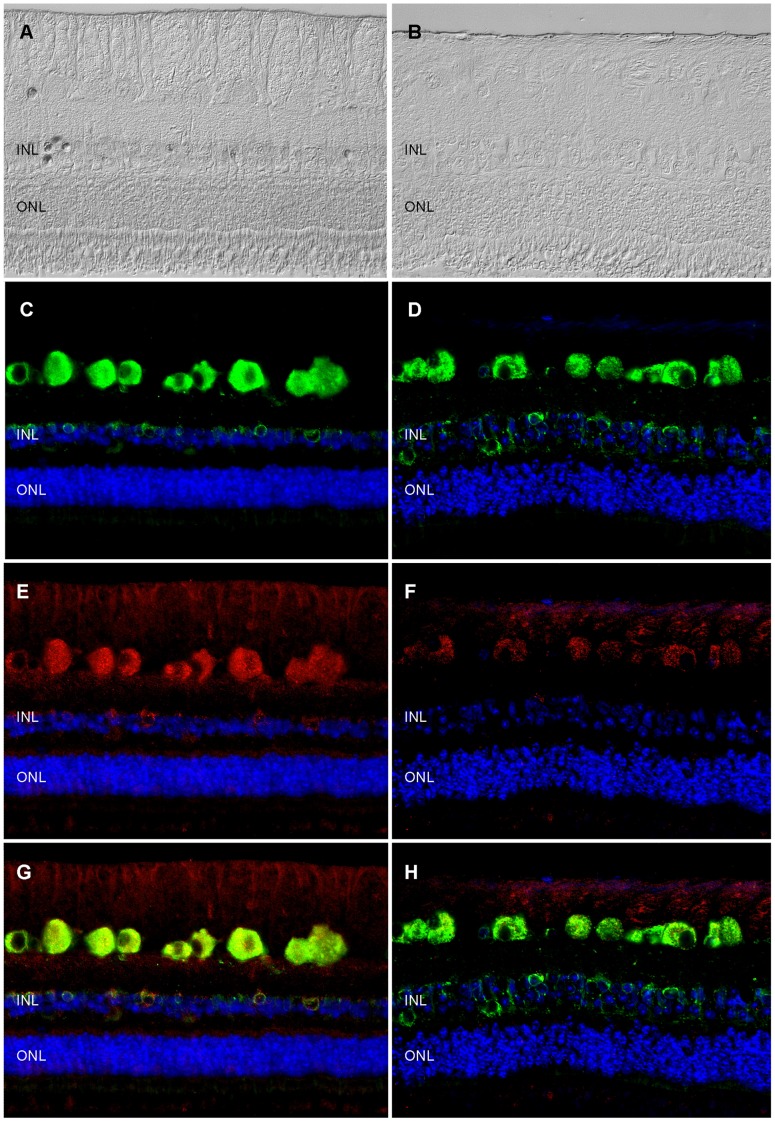
Immunohistochemical analysis of Syt1 expression changes in ERU retina. Left panels: representative healthy retina; right panels: representative ERU case. Differential interference contrast images of healthy (A) and ERU affected (B) retinal specimen demonstrating that in ERU state, normal retinal architecture is disturbed. GRP78 (green color), a marker staining retinal ganglion cells and a population of inner nuclear layer cells in equine retina was equally expressed in physiological (C) and ERU state (D). Synaptotagmin-1 (Syt1, red color) signal in healthy retina (E) was most prominent in retinal ganglion cell somata, their axons in the nerve fiber layer and in somata of a cell population in the inner nuclear layer, with additional staining foci in the outer and inner plexiform layer and photoreceptor outer segments, while ERU affected retinal sections (F), presented with a clearly reduced overall Syt1 signal. Overlay of GRP78 and Syt1 signals (G: healthy, H: ERU) indicated that in the ERU affected section, Syt1 expression is reduced, although structures expressing it in physiological state are still present. Cell nuclei were counterstained with DAPI (blue color).

Each protein has specific functions and is part of a biological network. Determining its expression levels in health and disease can provide information about whether dysregulations occur in diseased state, which in turn leads to a more detailed assessment of a protein's role in disease and a better understanding of pathogenesis-related mechanisms. Quantification by Western blot revealed that Syt1 expression was significantly (p≤0.05) reduced in ERU retinas (Fig. 3, right, black bar) to 24% (SD ±34%) of signal intensity in healthy retinal tissues (Fig. 3, left, white bar). Only in one ERU cases tested, Syt1 did not decrease, indicating that there are ERU affected individuals where retinal Syt1 expression is unchanged, recovers or decreases at varying points in the course of disease. However, the majority of ERU cases had significantly less Syt1 expression in retina. This indicates, that Syt1 is not stably expressed in retinal tissue unlike the already known ERU autoantigens, cellular retinaldehyde-binding protein, S-antigen and interphotoreceptor retinoid-binding protein [28]. Since Syt1 expression sites in equine retina were not described to date, we performed immunohistochemistry to localize Syt1 in horse retina (Fig. 4). In normal equine retina (Fig. 4A), Syt1-signaling was most prominent in retinal ganglion cells, followed by cells of the inner nuclear layer with additional staining occurring in nerve fiber layer, inner and outer plexiform layer and photoreceptor outer segments (Fig. 4D). The signal in retinal ganglion cells and cells of the inner nuclear layer was located in cell somata (Fig. 4D, Syt1 expression: red color). Aforementioned additional Syt1 expression foci in other layers were less clearly demarcated and of lower signal intensity (Fig. 4D). This staining pattern most likely reflects the meshwork structure in these layers, which are mostly composed of synapses and cellular processes reaching out from the adjacent layers containing the associated cell somata [24]. Due to the low extent of staining observed here, we hypothesize that in normal equine retina only a subset of synapses in the plexiform layers contains Syt1. Double-staining of retinal sections with antibodies to Syt1 and Glucose-related protein 78 (GRP78), a previously described marker for ganglion cells in the equine retina [29], confirmed expression of Syt1 in retinal ganglion cells by complete signal colocalization with Syt1 for this cell type in healthy samples (Fig. 4E, overlay of staining: yellow color). In addition to retinal ganglion cells, GRP78 stained a population of cells in the inner nuclear layer [29]. Interestingly, we observed a co-localization of GRP78 and Syt1 in most cells of the inner nuclear layer, although single-positive cells for both GRP78 and Syt1 occurred occasionally (Fig. 4G). Significant changes in Syt1 expression occurred in uveitic retinas (Fig. 4B) compared to healthy state. In ERU affected retinas, our most frequent observation was an overall reduction of Syt1 (Fig. 4F, representative ERU case), consistent with our findings from Western blot quantification (Fig. 3). Most structures showed a clear decrease of SYT1 staining in ERU, while remnant staining was observed primarily in retinal ganglion cells (Fig. 4F, representative ERU case). In plexiform layers, we observed an overall reduction of Syt1 signal. The number of Syt1-positive cells in the inner nuclear layer was generally decreased as well. Double-staining of GRP78 and Syt1 in ERU retinas confirmed an unchanged abundance of retinal ganglion cells in ERU, demonstrated by equal expression of GRP78 in both states (Fig. 4C and D, GRP 78 expression green). Contrary to this, we observed a clear decrease of Syt1 signal intensity in retinal ganglion cells and a decrease in Syt1 positive cells in the inner nuclear layer (Fig. 4F). Overlay of both signals in ERU specimen (Fig. 4H) confirmed that reduction of Syt1 expression in ERU affected retinas was not caused by complete destruction of Syt1-containing structures, since unchanged GRP78 expression demonstrated that respective structures were still present in ERU.

## Discussion

There is still a need to identify novel autoantigens in autoimmune diseases, because the emergence of novel autoantibody targets does not only complete our knowledge about the pathomechanisms of disease, but has also profound consequences for diagnosis and therapeutic approach to autoimmune diseases. This was recently shown for the autoantigen APQ4 in neuromyelitis optica, because detection of this cell membrane expressed antigen enabled stratification of patients with neuromyelitis optica from those with multiple sclerosis. This led to a new understanding of the factors behind clinical symptoms in this disease and subsequently paved the way for novel therapeutic strategies, which included blocking of the pathogenic binding of IgG to AQP4 and prevented its detrimental downstream effects [15], [16], [30]. The astrocytic water channel AQP4 is an integral membrane protein [15] and thus belongs to the subgroup of membrane proteins, where one would expect to find a large number of proteins targeted in autoimmune diseases, as these proteins are often found on cell boundaries which would be easily accessible for autoreactive immune cells and antibodies. In ERU, a spontaneous model for human autoimmune uveitis, a highly effective workflow for identification and validation of autoantigens was established in the past [5], however, no membrane protein was identified as autoantigen to date. It became clear that while unfractionated retinal tissue was an excellent antigenic source in previous screening experiments [3], [4], answering more specific questions required a specific adaptation of the experimental reading frame. In this study, prefractionation of retinal tissue as autoantigenic source for Western blot screenings was tested as a potential solution for this problem and proved to be an elementary step in order to specifically examine protein subgroups for potential novel autoantigens. In this case, glycoproteins, which, as previously mentioned, are predominantly found on cell membranes [9] were the focus point of examination. Results of our study clearly demonstrated, that enriching retinal glycoproteins via ConA affinity successfully reduced the complexity of retinal tissue as antigen pool in 1D Western blot screenings and enhanced the detectability of serum IgG reactions to low abundant glycoproteins (Fig. 1). This enabled us to identify and validate Syt1, a N-glycosylated transmembrane protein [31] and the first identified membrane protein target for ERU autoantibodies. While lectin affinity is widely taken to enrich glycoproteins for quantitative proteome analysis or differential proteomics [32], [33], the value of this fractionation method for the detection of novel autoantigens in autoimmune diseases was not fully exploited yet and might therefore lead to new and exciting insights. This is underlined by the fact that in the search for ERU autoantigens, we conducted successful studies in the past, which followed a basic proteomic workflow of identification and validation similar to the one applied in this study, but were based on the whole retinal proteome as autoantigenic source, without a preliminary enrichment step, and Syt1 never appeared as candidate in these studies [3], [5]. Syt1 is a protein integral to synaptic vesicle membranes, where it acts as sensor for the increase of cytoplasmatic Ca^2+^ that occurs after an opening of Ca^2+^ channels in response to an incoming action potential. Though the full details of this process are not yet understood, by interaction with the SNARE-complex, the binding of Ca^2+^ to Syt1 initiates the fusion of the vesicle membrane with the cell membrane, which is required for exocytosis of neurotransmitters into the synaptic cleft [26], [27], [34], [35], [36], [37]. Recent evidence suggested that Syt1 additionally has a positive regulatory effect on axonal branching during neuronal development [38]. Members of the synaptotagmin family were reported to be differentially expressed in a variety of neurodegenerative afflictions, including Parkinson's disease and Alzheimer's disease [39]. In patients affected with Alzheimer's disease, a downregulation of Syt1 was observed in different brain regions [39]. A dysregulation of hippocampal Syt1 expression was also reported in patients with mesial temporal lobe epilepsy, where it decreased and in the refractory group of patients of temporal lobe epilepsy, where it increased [39].

As autoantibody target, Syt1 was previously described in Lambert-Eaton myasthenic syndrome, an immune-mediated paraneoplastic disease affecting neuromuscular junctions, where it was suggested that Syt1 specific autoantibodies might interfere with presynaptic mechanisms and thus impair neuromuscular function [40], [41], [42], [43], [44], [45]. It is very likely that in ERU affected retina, the binding of autoreactive IgG to Syt1 also hinders previously mentioned physiological functions of Syt1 in neurotransmitter release, leading to a general dysregulation of functional circuits in the transmission of visual stimuli. According to our results in immunohistochemical analysis, this would affect a variety of cell types, including retinal ganglion cells, photoreceptors and a cell population in the inner nuclear layer. Via immunohistochemistry, the downregulation of Syt1 that we detected in ERU state was demonstrated to be independent of apparent destruction of physiological expression sites, thus we hypothesize that an active downregulation is the driving factor behind the reduction of Syt1 content in ERU affected retina specimens. Interestingly, downregulation of Syt1 in ERU as described in this study confirmed findings from a previous study analyzing differential protein expression of retinal membrane proteins. This result was not further verfied at that time, but already showed that Syt1 was downregulated in ERU retina [25]. Possible reasons for a Syt1 downregulation might be a reduced expression due to cellular stress during autoimmune attacks, a lacking ability to regenerate physiological levels of Syt1 that was destroyed or rendered ineffective by autoantibody binding or a consequence of dysregulated upstream factors influencing Syt1 expression which were not detected yet. While these hypotheses do not exclude each other and might even occur concurrently, the last mentioned possibility is probably the one that is most compelling to explore in future studies, as e.g. the synaptic vesicle protein SV2B, which interacts with Syt1, did not only influence expression levels of Syt1, but also of several other synaptic vesicle proteins in retinal tissue [46]. In addition, our findings in immunohistochemistry were especially interesting, as they corroborated previous evidence pointing to species specific differences of retinal Syt1 expression, setting equine retina apart from mouse retina, where in immunohistochemistry, Syt1 was observed mostly in the plexiform layers [46], [47]. Koontz and Hendrickson described a somatic Syt1 signal in primate retina and attributed it to somata and fibers of displaced amacrine cells for the ganglion cell layer, and in the inner nuclear layer to bipolar cell axons and somata and amacrine cell somata [48]. However, due to the previously mentioned colocalization of Syt1 with GRP78 and the signal in the nerve fiber layer, evidence points to retinal ganglion cells as main site of Syt1 expression in the equine retinal ganglion cell layer. As Syt1 was shown to be part of the photoreceptor outer segment proteome [49], [50], we conclude that the signal we observed in photoreceptor outer segments is specific. Interestingly, Synaptotagmin-1 is also expressed in other neuronal tissues apart from the retina, for example in pinealocytes [51]. Other antigens in ERU [52] are also known to be expressed in retina and pineal gland, for example S-Antigen [53], [54] and IRBP [55]. In ERU patients, a concurrent pinealitis is known to develop [56], and as it is expressed in both retina and pineal gland, Synaptotagmin-1 as autoantigen might contribute to pineal immunopathology in ERU. As Synaptotagmin-1 is an autoantigen which is not exclusively expressed in retina, another interesting question that will be approached in future studies is whether there are any local intraocular factors that cause an autoimmune response against this antigen which is detrimental locally, but not systemically. Autoimmunity against systemic antigens that led to organ-specific symptoms only was already described in a mouse model of rheumatoid arthritis [57]. After immunization with the ubiquitous protein Glucose-6-Phosphate Isomerase, genetically unaltered mice developed joint-specific symptoms [57]. Reasons for this mechanisms are still unclear, but it was proposed that locally increased concentration of G6PI in joints might contribute to the initiation of the immune response against this protein and that local, joint-specific factors like regulation of cytokine expression might lead to development of a pathogenic response [57]. In order to validate the identification of Syt1 as autoantigen and to assess the prevalence of anti Syt1 IgG serum reactions, indirect ELISA was performed. We were able to validate our identification from Western blot screening and observed that serum IgG indeed target Syt1, and that they have a prevalence of 56% in the ERU group (Fig. 2B), with significantly more positive reactions in the ERU group (* = p≤0.05) compared to healthy control sera. While reactions occurred in both groups, IgG autoantibodies to Syt1 in the healthy and ERU group might differ in their properties, for example their affinity or the epitope that is bound. Another important factor to consider is that equine IgG comprise seven subclasses [58]. A difference in subclass of Syt-1 targeting autoantibodies might be decisive in harmless versus harmful autoreactivity, as it is known that IgG can recruit pro-inflammatory or anti-inflammatory pathways, depending on their subclass or post-translational modifications [59], [60], as it was observed for example in rheumatoid arthritis, where a changed glycosylation pattern of IgG is associated with pathogenesis [61]. For equine IgG, only limited information about their functions is available. It is already known that IgG1, IgG3, IgG4, IgG5 and IgG7 are probably able to interact with Fc receptors on immune effector cells and that IgG1, IgG3, IgG4 and IgG7 are able to bind complement C1q [58]. Thus, functional differences might also be of interest for ERU research, as they might explain why autoreactivites against a specific protein are harmless in some animals, but associated with ERU in others. Thus, future studies should ideally aim at a thorough comparison of IgG properties in ERU affected individuals versus healthy individuals. The question whether the seven equine IgG subclasses have a role in determining whether an individual is susceptible to ERU or whether they influence the course of disease needs to be answered in the future. Unfortunately, a lack of specific tools is currently preventing studies of this aspect, as currently available reagents are specific for the obsolete classification IgGa, IgGb, IgGc and IgGT, and are not able to provide a valid distinction between all seven IgG isotypes of the horse [58].

In addition, autoreactive sera IgG specific for certain targets are proven biomarkers in some autoimmune diseases [15] and were shown to possess important predictive value in several diseases, for example type I diabetes [62]. In a large scale studies with children, a specific constellation of IgG autoantibodies indicated development of type I diabetes later in life, these predictive autoantibodies were present for several years before diabetes developed [62]. ERU has a high prevalence of 10% [63] and it is possible that sera tested in our ELISA were accidentally derived from individuals that were healthy at the time of sampling, but are at risk of developing ERU in the future. One of the most important questions that will have to be answered in future studies is whether Synapotagmin-1 as autoantigen is directly pathogenic in ERU. This could be answered e.g. by testing its ability to induce experimental uveitis in horses via immunization with this protein. Having learned from our results that Syt1 is a significant factor in ERU, further studies will be necessary in order to assess the physiological role of Syt1 in equine retina and its precise role in ERU pathology.

## Conclusions

Results of this study will have several implications for future projects. First, we demonstrated that a pre-fractionation of autoantigenic targets is an effective preliminary step to reduce sample complexity and to enrich for interesting protein subgroups. Glycoprotein isolation by ConA affinity proved a suitable method to search for membrane-bound autoantigens. Further adaptations of these experimental settings might enable the examination of other retinal protein fractions, for example the O-glycosylated fraction instead of the N-glycosylated fraction that was enriched by using Con-A affinity, and lead to the discovery of even more novel autoantigens The identification of Syt1 as novel membrane autoantigen with a high autoantibody prevalence in the ERU group strongly suggests that an impairment of neurotransmitter release plays a role in ERU and its reduced expression in ERU affected retina indicates an active downregulation. Interestingly, Syt1 might not be de-regulated in all ERU cases, pointing to a need for further investigation of factors that might be associated with Syt1 downregulation and anti Syt1 autoantibodies.

## References

[1] Deeg CA, Hauck SM, Amann B, Pompetzki D, Altmann F, et al (2008) Equine recurrent uveitis—a spontaneous horse model of uveitis. Ophthalmic Res 40: 151–153. 18421230 10.1159/000119867

[2] Degroote RL, Hauck SM, Kremmer E, Amann B, Ueffing M, et al (2012) Altered expression of talin 1 in peripheral immune cells points to a significant role of the innate immune system in spontaneous autoimmune uveitis. J Proteomics 75: 4536–4544. 22306886 10.1016/j.jprot.2012.01.023

[3] Zipplies JK, Hauck SM, Eberhardt C, Hirmer S, Amann B, et al (2012) Miscellaneous vitreous-derived IgM antibodies target numerous retinal proteins in equine recurrent uveitis. Vet Ophthalmol doi:10.1111/j.1463-5224.2012.01010.x. 10.1111/j.1463-5224.2012.01010.x 22432720

[4] Swadzba ME, Hirmer S, Amann B, Hauck SM, Deeg CA (2012) Vitreal IgM autoantibodies target neurofilament medium in a spontaneous model of autoimmune uveitis. Invest Ophthalmol Vis Sci 53: 294–300. 22199250 10.1167/iovs.11-8734

[5] Deeg CA, Pompetzki D, Raith AJ, Hauck SM, Amann B, et al (2006) Identification and functional validation of novel autoantigens in equine uveitis. Mol Cell Proteomics 5: 1462–1470. 16690753 10.1074/mcp.M500352-MCP200

[6] Gauba V, Grunewald J, Gorney V, Deaton LM, Kang M, et al (2011) Loss of CD4 T-cell-dependent tolerance to proteins with modified amino acids. Proc Natl Acad Sci U S A 108: 12821–12826. 21768354 10.1073/pnas.1110042108 PMC3150954

[7] Petersen J, Purcell AW, Rossjohn J (2009) Post-translationally modified T cell epitopes: immune recognition and immunotherapy. J Mol Med (Berl) 87: 1045–1051. 19763524 10.1007/s00109-009-0526-4

[8] Marino K, Bones J, Kattla JJ, Rudd PM (2010) A systematic approach to protein glycosylation analysis: a path through the maze. Nat Chem Biol 6: 713–723. 20852609 10.1038/nchembio.437

[9] Josic D, Clifton JG (2007) Mammalian plasma membrane proteomics. Proteomics 7: 3010–3029. 17654460 10.1002/pmic.200700139

[10] Deeg CA, Ehrenhofer M, Thurau SR, Reese S, Wildner G, et al (2002) Immunopathology of recurrent uveitis in spontaneously diseased horses. Exp Eye Res 75: 127–133. 12137758 10.1006/exer.2002.2011

[11] Deeg CA (2008) Ocular immunology in equine recurrent uveitis. Vet Ophthalmol 11 Suppl 1: 61–65. 10.1111/j.1463-5224.2008.00625.x 19046272

[12] Deeg CA, Altmann F, Hauck SM, Schoeffmann S, Amann B, et al (2007) Down-regulation of pigment epithelium-derived factor in uveitic lesion associates with focal vascular endothelial growth factor expression and breakdown of the blood-retinal barrier. Proteomics 7: 1540–1548. 17407186 10.1002/pmic.200600795

[13] Caspi RR (2010) A look at autoimmunity and inflammation in the eye. J Clin Invest 120: 3073–3083. 20811163 10.1172/JCI42440 PMC2929721

[14] Levy RA, de Andrade FA, Foeldvari I (2011) Cutting-edge issues in autoimmune uveitis. Clin Rev Allergy Immunol 41: 214–223. 21913066 10.1007/s12016-011-8267-x

[15] Papadopoulos MC, Verkman A (2012) Aquaporin 4 and neuromyelitis optica. Lancet Neurol 11: 535–544. 22608667 10.1016/S1474-4422(12)70133-3 PMC3678971

[16] Ratelade J, Verkman AS (2012) Neuromyelitis Optica: Aquaporin-4 Based Pathogenesis Mechanisms and New Therapies. Int J Biochem Cell Biol 44: 1519–1530. 22713791 10.1016/j.biocel.2012.06.013 PMC3676174

[17] Lennon VA, Wingerchuk DM, Kryzer TJ, Pittock SJ, Lucchinetti CF, et al (2004) A serum autoantibody marker of neuromyelitis optica: distinction from multiple sclerosis. Lancet 364: 2106–2112. 15589308 10.1016/S0140-6736(04)17551-X

[18] Liu XQ, Kobayashi H, Jin ZB, Wada A, Nao IN (2007) Differential expression of Kir4.1 and aquaporin 4 in the retina from endotoxin-induced uveitis rat. Mol Vis 13: 309–317. 17356517 PMC2642914

[19] Zhao M, Bousquet E, Valamanesh F, Farman N, Jeanny JC, et al (2011) Differential regulations of AQP4 and Kir4.1 by triamcinolone acetonide and dexamethasone in the healthy and inflamed retina. Invest Ophthalmol Vis Sci 52: 6340–6347. 21724913 10.1167/iovs.11-7675

[20] Eberhardt C, Amann B, Feuchtinger A, Hauck SM, Deeg CA (2011) Differential expression of inwardly rectifying K+ channels and aquaporins 4 and 5 in autoimmune uveitis indicates misbalance in Muller glial cell-dependent ion and water homeostasis. Glia 59: 697–707. 21305615 10.1002/glia.21139

[21] Becker JW, Reeke GN Jr, Wang JL, Cunningham BA, Edelman GM (1975) The covalent and three-dimensional structure of concanavalin A. III. Structure of the monomer and its interactions with metals and saccharides. J Biol Chem 250: 1513–1524. 1112815

[22] Naim HY, Koblet H (1992) Asparagine-linked oligosaccharides of Semliki Forest virus grown in mosquito cells. Arch Virol 122: 45–60. 1729985 10.1007/BF01321117

[23] Hauck SM, Schoeffmann S, Amann B, Stangassinger M, Gerhards H, et al (2007) Retinal Mueller glial cells trigger the hallmark inflammatory process in autoimmune uveitis. J Proteome Res 6: 2121–2131. 17444670 10.1021/pr060668y

[24] Ehrenhofer MC, Deeg CA, Reese S, Liebich HG, Stangassinger M, et al (2002) Normal structure and age-related changes of the equine retina. Vet Ophthalmol 5: 39–47. 11940247 10.1046/j.1463-5224.2002.00210.x

[25] Hauck SM, Dietter J, Kramer RL, Hofmaier F, Zipplies JK, et al (2010) Deciphering membrane-associated molecular processes in target tissue of autoimmune uveitis by label-free quantitative mass spectrometry. Mol Cell Proteomics 9: 2292–2305. 20601722 10.1074/mcp.M110.001073 PMC2953921

[26] Ariel P, Ryan TA (2012) New insights into molecular players involved in neurotransmitter release. Physiology (Bethesda) 27: 15–24. 22311967 10.1152/physiol.00035.2011 PMC3703655

[27] de Wit H, Walter AM, Milosevic I, Gulyas-Kovacs A, Riedel D, et al (2009) Synaptotagmin-1 docks secretory vesicles to syntaxin-1/SNAP-25 acceptor complexes. Cell 138: 935–946. 19716167 10.1016/j.cell.2009.07.027

[28] Deeg CA, Hauck SM, Amann B, Kremmer E, Stangassinger M, et al (2007) Major retinal autoantigens remain stably expressed during all stages of spontaneous uveitis. Mol Immunol 44: 3291–3296. 17467057 10.1016/j.molimm.2007.02.027

[29] Deeg CA, Amann B, Hauck SM, Kaspers B (2006) Defining cytochemical markers for different cell types in the equine retina. Anat Histol Embryol 35: 412–415. 17156097 10.1111/j.1439-0264.2006.00722.x

[30] Lennon VA, Kryzer TJ, Pittock SJ, Verkman AS, Hinson SR (2005) IgG marker of optic-spinal multiple sclerosis binds to the aquaporin-4 water channel. J Exp Med 202: 473–477. 16087714 10.1084/jem.20050304 PMC2212860

[31] Han W, Rhee JS, Maximov A, Lao Y, Mashimo T, et al (2004) N-glycosylation is essential for vesicular targeting of synaptotagmin 1. Neuron 41: 85–99. 14715137 10.1016/s0896-6273(03)00820-1

[32] Shamsi KS, Pierce A, Ashton AS, Halade DG, Richardson A, et al (2012) Proteomic Screening of Glycoproteins in Human Plasma for Frailty Biomarkers. J Gerontol A Biol Sci Med Sci doi:10.1093/gerona/glr1224. 10.1093/gerona/glr224 PMC3403859 22219522

[33] Wang Y, Ao X, Vuong H, Konanur M, Miller FR, et al (2008) Membrane glycoproteins associated with breast tumor cell progression identified by a lectin affinity approach. J Proteome Res 7: 4313–4325. 18729497 10.1021/pr8002547 PMC2630886

[34] Vrljic M, Strop P, Ernst JA, Sutton RB, Chu S, et al (2010) Molecular mechanism of the synaptotagmin-SNARE interaction in Ca2+-triggered vesicle fusion. Nat Struct Mol Biol 17: 325–331. 20173762 10.1038/nsmb.1764 PMC2928146

[35] Vennekate W, Schroder S, Lin CC, van den Bogaart G, Grunwald M, et al (2012) Cis- and trans-membrane interactions of synaptotagmin-1. Proc Natl Acad Sci U S A 109: 11037–11042. 22711810 10.1073/pnas.1116326109 PMC3390864

[36] McNeil BD, Wu LG (2009) Location matters: synaptotagmin helps place vesicles near calcium channels. Neuron 63: 419–421. 19709623 10.1016/j.neuron.2009.08.001 PMC4888869

[37] Chapman ER (2008) How does synaptotagmin trigger neurotransmitter release? Annu Rev Biochem 77: 615–641. 18275379 10.1146/annurev.biochem.77.062005.101135

[38] Greif KF, Asabere N, Lutz GJ, Gallo G (2012) Synaptotagmin-1 promotes the formation of axonal filopodia and branches along the developing axons of forebrain neurons. Dev Neurobiol doi:10.1002/dneu.22033. 10.1002/dneu.22033 22589224

[39] Glavan G, Schliebs R, Zivin M (2009) Synaptotagmins in neurodegeneration. Anat Rec (Hoboken) 292: 1849–1862. 19943339 10.1002/ar.21026

[40] David P, Martin-Moutot N, Leveque C, el Far O, Takahashi M, et al (1993) Interaction of synaptotagmin with voltage gated calcium channels: a role in Lambert-Eaton myasthenic syndrome? Neuromuscul Disord 3: 451–454. 8186692 10.1016/0960-8966(93)90095-2

[41] Takamori M, Komai K, Iwasa K (2000) Antibodies to calcium channel and synaptotagmin in Lambert-Eaton myasthenic syndrome. Am J Med Sci 319: 204–208. 10768604 10.1097/00000441-200004000-00002

[42] Takamori M (2008) Lambert-Eaton myasthenic syndrome: search for alternative autoimmune targets and possible compensatory mechanisms based on presynaptic calcium homeostasis. J Neuroimmunol 201–202: 145–152. 10.1016/j.jneuroim.2008.04.040 18653248

[43] Takamori M (2004) Lambert-Eaton myasthenic syndrome as an autoimmune calcium channelopathy. Biochem Biophys Res Commun 322: 1347–1351. 15336982 10.1016/j.bbrc.2004.08.040

[44] Martin-Moutot N, el Far O, Leveque C, David P, Marqueze B, et al (1993) Synaptotagmin: a Lambert-Eaton myasthenic syndrome antigen that associates with presynaptic calcium channels. J Physiol Paris 87: 37–41. 8305896 10.1016/0928-4257(93)90022-l

[45] Takamori M, Hamada T, Komai K, Takahashi M, Yoshida A (1994) Synaptotagmin can cause an immune-mediated model of Lambert-Eaton myasthenic syndrome in rats. Ann Neurol 35: 74–80. 8285596 10.1002/ana.410350112

[46] Morgans CW, Kensel-Hammes P, Hurley JB, Burton K, Idzerda R, et al (2009) Loss of the Synaptic Vesicle Protein SV2B results in reduced neurotransmission and altered synaptic vesicle protein expression in the retina. PLoS One 4: e5230. 19381277 10.1371/journal.pone.0005230 PMC2667261

[47] Berntson AK, Morgans CW (2003) Distribution of the presynaptic calcium sensors, synaptotagmin I/II and synaptotagmin III, in the goldfish and rodent retinas. J Vis 3: 274–280. 12803536 10.1167/3.4.3

[48] Koontz MA, Hendrickson AE (1993) Comparison of immunolocalization patterns for the synaptic vesicle proteins p65 and synapsin I in macaque monkey retina. Synapse 14: 268–282. 8248851 10.1002/syn.890140405

[49] Kwok MC, Holopainen JM, Molday LL, Foster LJ, Molday RS (2008) Proteomics of photoreceptor outer segments identifies a subset of SNARE and Rab proteins implicated in membrane vesicle trafficking and fusion. Mol Cell Proteomics 7: 1053–1066. 18245078 10.1074/mcp.M700571-MCP200 PMC2424196

[50] Kiel C, Vogt A, Campagna A, Chatr-aryamontri A, Swiatek-de Lange M, et al (2011) Structural and functional protein network analyses predict novel signaling functions for rhodopsin. Mol Syst Biol 7: 551. 22108793 10.1038/msb.2011.83 PMC3261702

[51] Redecker P (1996) Synaptotagmin I, synaptobrevin II, and syntaxin I are coexpressed in rat and gerbil pinealocytes. Cell Tissue Res 283: 443–454. 8593674 10.1007/s004410050555

[52] Deeg CA, Kaspers B, Gerhards H, Thurau SR, Wollanke B, et al (2001) Immune responses to retinal autoantigens and peptides in equine recurrent uveitis. Invest Ophthalmol Vis Sci 42: 393–398. 11157872

[53] Collin JP, Mirshahi M, Brisson P, Falcon J, Guerlotte J, et al (1986) Pineal-retinal molecular relationships: distribution of “S-antigen” in the pineal complex. Neuroscience 19: 657–666. 3534623 10.1016/0306-4522(86)90288-5

[54] Mirshahi M, Faure JP, Brisson P, Falcon J, Guerlotte J, et al (1984) S-antigen immunoreactivity in retinal rods and cones and pineal photosensitive cells. Biol Cell 52: 195–198. 6241493 10.1111/j.1768-322x.1985.tb00336.x

[55] Wiggert B, Lee L, Rodrigues M, Hess H, Redmond TM, et al (1986) Immunochemical distribution of interphotoreceptor retinoid-binding protein in selected species. Invest Ophthalmol Vis Sci 27: 1041–1049. 3721783

[56] Kalsow CM, Dubielzig RR, Dwyer AE (1999) Immunopathology of pineal glands from horses with uveitis. Invest Ophthalmol Vis Sci 40: 1611–1615. 10359346

[57] Schubert D, Maier B, Morawietz L, Krenn V, Kamradt T (2004) Immunization with glucose-6-phosphate isomerase induces T cell-dependent peripheral polyarthritis in genetically unaltered mice. J Immunol 172: 4503–4509. 15034067 10.4049/jimmunol.172.7.4503

[58] Lewis MJ, Wagner B, Woof JM (2008) The different effector function capabilities of the seven equine IgG subclasses have implications for vaccine strategies. Mol Immunol 45: 818–827. 17669496 10.1016/j.molimm.2007.06.158 PMC2075531

[59] Bohm S, Schwab I, Lux A, Nimmerjahn F (2012) The role of sialic acid as a modulator of the anti-inflammatory activity of IgG. Semin Immunopathol 34: 443–453. 22437760 10.1007/s00281-012-0308-x

[60] Lux A, Aschermann S, Biburger M, Nimmerjahn F (2010) The pro and anti-inflammatory activities of immunoglobulin G. Ann Rheum Dis 69 Suppl 1: i92–96. 19995755 10.1136/ard.2009.117101

[61] Troelsen LN, Jacobsen S, Abrahams JL, Royle L, Rudd PM, et al (2012) IgG glycosylation changes and MBL2 polymorphisms: associations with markers of systemic inflammation and joint destruction in rheumatoid arthritis. J Rheumatol 39: 463–469. 22247351 10.3899/jrheum.110584

[62] Steck AK, Johnson K, Barriga KJ, Miao D, Yu L, et al (2011) Age of islet autoantibody appearance and mean levels of insulin, but not GAD or IA-2 autoantibodies, predict age of diagnosis of type 1 diabetes: diabetes autoimmunity study in the young. Diabetes Care 34: 1397–1399. 21562325 10.2337/dc10-2088 PMC3114355

[63] Spiess BM (2010) Equine recurrent uveitis: the European viewpoint. Equine Vet J 42 Suppl 37: 50–56. 10.1111/j.2042-3306.2010.tb05635.x 20939167

